# *Porphyromonas gingivalis* promotes the formation and development of type 2 diabetes mellitus complicated with cognitive impairment by regulating gut microbiota and metabolic pathways

**DOI:** 10.1186/s10020-025-01342-y

**Published:** 2025-09-26

**Authors:** Xin Liu, Keke Che, Qiaoli Li, Xiuli Wu, Dong Zeng, Xiaoli Du, Shanglan Qing

**Affiliations:** 1 Department of Stomatology, The Thirteenth People’s Hospital of Chongqing, Chongqing, 400053 China; 2 Department of Pharmacy, Chongqing General Hospital, Chongqing, 400014 China; 3 Department of Stomatology, Chongqing General Hospital, Chongqing, 400014 China

**Keywords:** *Porphyromonas gingivalis*, Cognitive impairment, Type 2 diabetes mellitus, Gut microbiota, Metabolic pathways

## Abstract

**Background:**

Type 2 diabetes mellitus (T2DM) is associated with a series of chronic complications, of which cognitive impairment is a common complication of the central nervous system. *Porphyromonas gingivalis* (Pg), a gram-negative anaerobic bacillus with non-glycolytic sugar, is thought to be associated with the aggravation of diabetes, diabetes complications, and senile dementia. This study aimed to explore the role and mechanism of Pg in the development of cognitive impairment in T2DM.

**Methods:**

Cognitive ability, hippocampal neuron damage, synaptic plasticity, and microglial inflammation were analyzed in mice. Furthermore, the changes of microflora in the colon contents, and the content of short-chain fatty acids (SCFAs) in serum, and the expression of SCFA receptor Olfr78 in the hippocampus were analyzed.

**Results:**

Pg promoted cognitive impairment, aggravated hippocampal neuron damage, reduced synaptic plasticity, and promoted microglial inflammatory response in T2DM mice. Gut microbiota sequencing and serum metabolomics results showed that Pg promoted gut microbiota disturbance in T2DM mice, affected the serum content of SCFAs, and reduced the expression of Olfr78 in the hippocampal tissue of T2DM mice.

**Conclusion:**

Pg may mediate the gut-brain axis through gut microbiota and its metabolic pathway, and participate in cognitive dysfunction of T2DM.

## Introduction

Diabetes is one of the most common metabolic diseases, characterized by chronic hyperglycemia and dyslipidemia, and is the third noncommunicable disease that threatens people’s health(Refardt et al. 2020). About 400 million people worldwide have diabetes, and the number continues to increase, with type 2 diabetes mellitus (T2DM) accounting for the vast Majority, about 90%(Zheng et al. 2018). Abnormal glucose and insulin metabolism in T2DM patients can damage multiple organ systems, and the central nervous system (CNS) is one of the most commonly involved organs(Zhang et al. 2020a, b). The impact of T2DM on the CNS is mainly manifested as the decline of learning and memory function, the decline of language, understanding, judgment, and problem-solving ability, and the inability to take care of themselves in serious cases, which is considered to be the T2DM-associated cognitive dysfunction(Ehtewish et al. 2022; Liu et al. 2020). The survey showed that the incidence of cognitive impairment in T2DM was higher than that in non-diabetic patients, and 60%−70% of T2DM patients had reduced cognitive function(Damanik and Yunir 2021). Between 1/10 and 1/15 cases of dementia worldwide can be attributed to T2DM(Selman et al. 2022; Li and Huang 2016). T2DM-related cognitive dysfunction not only leads to a serious decline in self-care ability, but also increases the incidence and mortality of related diseases in T2DM patients(Ma et al. 2022). Although blood glucose can be effectively controlled clinically, it cannot completely prevent the development of cognitive impairment. The specific mechanism of cognitive dysfunction in T2DM is still unclear, and it is important to study its pathogenesis.

*Porphyromonas gingivalis* (Pg) is a gram-negative oral anaerobic bacteria, which is currently considered the main cause of periodontitis(Mysak et al. 2014). Studies have found that it can enter the intestine and cause changes in the composition of gut microbiota, leading to inflammatory reactions in other organs, thereby increasing the risk of systemic diseases characterized by low-grade inflammation(Zhang et al. 2023). In addition, studies have shown that Pg can survive in organs than the mouth, for example, live Pg has been detected in human atherosclerotic plaque tissue(Kozarov et al. 2005) and mouse lungs(Benedyk et al. 2016). Pg was also isolated from human aortic endothelial cells(Yamatake et al. 2007), human pancreatic tumor cells(Gnanasekaran et al. 2020), and human bone marrow dendritic cells(El-Awady et al. 2015). There is evidence that Pg is found in the brain and cerebrospinal fluid of Alzheimer’s disease (AD) patients, and Pg can promote its development by inducing neuroinflammation(Costa et al. 2021). Pg has the ability to invade distant tissues, participate in the onset and progression of systemic diseases, and may be related to CNS(Zhang et al. 2020a, b). In addition, studies have confirmed that Pg can affect T2DM through the oral-intestinal pathway(Carter et al. 2017), but its role in T2DM-related cognitive impairment and the specific mechanism are not fully understood.

In this study, a high-fat diet combined with streptozotocin (STZ) was used to construct a T2DM mouse model. The effects of Pg on the cognitive function of T2DM mice were observed from behavioral studies, and the effects of Pg on hippocampal neuron injury and synaptic plasticity in T2DM mice were observed by morphology, the inflammatory responses of microglia were detected, and the changes of gut microbiota and its metabolites were analyzed, so as to explore the role and possible mechanism of Pg on T2DM-related cognitive dysfunction.

## Materials and methods

### Animals experimental design

**Fig. 1:**
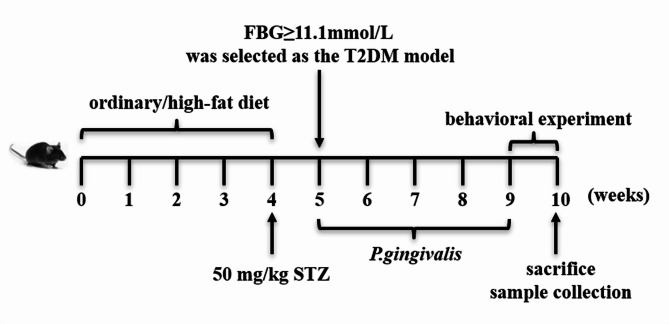
Animal testing arrangements

Forty SPF Male 4-week-old C57BL/6J mice were purchased from Chengdu Dossy Animal Experimental Co., LTD (Chengdu, China). All experiments were approved by the Ethics Committee of Chongqing General Hospital. The mice were randomly divided into normal and T2DM model groups. In the first four weeks, mice in the normal group were fed an ordinary diet, and T2DM model mice were fed a high-fat diet (D12492, Biopike, China) for 4 weeks. After fasting overnight, mice in the model group were intraperitoneally injected with 50 mg/kg streptozotocin (STZ, S8050, Solarbio, China), while mice in the normal group were intraperitoneally injected with the same amount of citric acid buffer. After 1 week, fasting plasma glucose (FBG) in the mice was detected, and FBG ≥ 11.1 mmol/L was confirmed as successful T2DM modeling. The model group was further divided into T2DM group and Pg + T2DM group, and the normal group was divided into control group and Pg control group (Pg), with 6 mice in each group. The Pg group and the Pg + T2DM group were injected with 200 µL 1.0 × 10^8^ CFU Pg solution (ATCC33277, Jinbio, China) in the tail vein of normal and T2DM mice, respectively(Sasaki et al. 2018). The administration was given once on Monday, Wednesday, and Friday each week, 3 times a week for 4 weeks, and the other groups were injected with 200 µL PBS. Random blood glucose was monitored weekly, oral glucose tolerance tests (OGTT) and insulin tolerance tests (ITT) were conducted, and the Morris water maze test and novel object recognition were performed to evaluate cognitive function after the experiment. After the behavioral experiment, the mice were sacrificed the next day for cervical dislocation, and brain tissue, blood, and colon contents were collected for follow-up experiments. The experimental arrangement was shown in Fig. 1.

## Glucose tolerance and insulin tolerance tests

Before the experiments, the mice were fasted for 16 h, and their baseline blood glucose level was measured. In the OGTT, 2 g/kg glucose solution was administered by gavage, and in the ITT, 0.5 U/kg insulin was injected via the tail vein. Blood glucose concentrations were measured at 15, 30, 60, 90, and 120 min after different treatments, and the blood glucose-time curves were plotted, and the area under curve (AUC) was calculated.

## Behavioral experiment

After different treatments, the Morris water Maze and the new object recognition experiment were carried out. The water Maze test Mainly includes the training stage and the experiment stage. The pool is divided into four quadrants, and the platform is fixed in the second quadrant. The day before the test, the mice were put into the pool to swim for 2 min and get familiar with the environment. The training stage lasted for 4 days, Mainly testing the learning ability of mice. Mice were put into the water from each quadrant with their heads facing the pool wall, and the swimming track of mice was recorded by the video acquisition system. When the mice found the platform and stabilized on the platform for 5 s, the experiment was over. If the mice did not find the platform within 60 s, the escape latency was 60 s, and then the mice were placed on the platform for 20 s. The fifth day is the experimental stage, the original platform is removed, and the total test time is 2 min. During the experiment, the video acquisition system recorded the mice’s latency time and the number of times they crossed the original platform placement, and calculated the average swimming speed.

The novel object recognition experiment lasted for four days, and the first two days were for habituation, mice were placed in the test box to get familiar with the environment in the box for 5 min. On the third day of training, two identical objects were put into the test box, and the mice were allowed to freely explore for 10 min in the box, and the total exploration time of the two objects was recorded. On the fourth day, an old object was randomly replaced by a new object, and the mice were allowed to enter the test box to explore freely for 5 min. The novel object recognition index (NOI) of the mice in the detection stage was analyzed. NOI (%) = (time spent exploring new objects - time spent exploring old objects)/total exploration time ×100. Before each mouse was tested, the test chamber was wiped with 75% alcohol to avoid the odor left by the previous mouse interfering with the experimental results.

## Blood biochemical index detection

During the experiment, the dynamic changes of random blood glucose in mice were analyzed by glucose meter. After the experiment, blood samples from mice were collected, and serum samples were obtained by centrifugation at 3000 rpm for 15 min. The blood lipids serum total cholesterol (TC), triglyceride (TG), low-density lipoprotein-cholesterol (LDL-C), and high-density lipoprotein-cholesterol (HDL-C) were examined by blood biochemical instrument. All operations are carried out in accordance with the reagent instructions.

## Hematoxylin-eosin staining (HE)

The brain tissues of mice in each group were collected, dehydrated by gradient alcohol after fixation, paraffin embedding, and sliced. After the sections were dewaxed, HE staining was performed, and the images of the sections were collected by the microscope (BA210Digital, Panthera, China) to observe the pathological changes in the brain.

### Nissl staining

The brain tissue sections were treated with 3-Aminopropyltriethoxysilane (APES) to prevent them from falling off and being dewaxed, then stained with 1% toluidine blue aqueous solution (G1032, Servicebio, China) preheated to 50℃ at 56℃ for 20 min, washed with distilled water and soaked in 70% alcohol for 1 min. After 95% alcohol was used for differentiation, gradient alcohol was dehydrated, xylene transparent, and neutral gum was sealed. Finally, the slices were observed and photographed under a microscope (BA210Digital, Panthera, China). The Nissl bodies in the hippocampal CA1 region were counted in three 400× field images collected from each sample. The Nissl bodies were dark blue and the background was colorless.

## TUNEL staining

After dewaxing the paraffin sections of brain tissue, they were repaired with citric acid (ZLI-9065, zsbio, China) for 8 min by microwave, incubated with TUNEL incubation solution (1168479590, Roche Group, Switzerland) at 37℃ for 1 h, then stained nucleus with DAPI for 15 min, rinsed with PBS, sealed with glycerin gelatin, observed and photographed by fluorescence microscope (Pannoramic 250, 3DHISTECH, Hungary). For each section, 400× field images of three regions were randomly collected. Normal cells and apoptotic cells in the images were counted respectively using Image Pro Plus 6.0 software, and the percentage of apoptotic cells was calculated as apoptotic cells/(normal cells + apoptotic cells). Normal cell nuclei show blue light, while apoptotic cell nuclei show green light and overlap with the blue light.

## Transmission electron microscope

The mouse hippocampus was prefixed with 3% glutaraldehyde, then postfixed with 1% osmium tetroxide, dehydrated with gradient acetone, and embedded with Epon812. Ultrathin sections of 60 ~ 90 nm were prepared by ultrathin section mechanism (UC7rt, LEICA, Germany), stained with uranium acetate for 10 ~ 15 min, then stained with lead citrate for 1 ~ 2 min, observed and photographed by transmission electron microscope (JEM-1400FLASH, JEOL, Japan). The total length of the postsynaptic density (PSD) was measured along the bending edge of the PSD using Image Pro Plus 6.0 software, and the maximum thickness was measured perpendicular to the PSD film plane.

### Immunofluorescent staining

The SYN, Iba1, and Olfr78 in the CA1 region of the mouse hippocampus were detected by immunofluorescence. After paraffin sections were dewaxed to water, the antigen was repaired by microwave oven with citrate buffer (pH 6.0) for 20 min. The endogenous peroxidase was blocked when incubated with 3% hydrogen peroxide at room temperature for 25 min without Light. After bovine serum albumin was blocked for 30 min, the primary antibodies SYN (1:100; IPB6304, Baijia, China), Iba1 (1:100; GB12105-100, Servicebio, China) and Olfr78 (1:100; PA5-99482, ThermoFisher, USA) were incubated at 4 °C overnight, and the secondary antibodies FITC conjugated Goat Anti-Rabbit (GB22303, Servicebio, China) and Cy3 conjugated Goat Anti-mouse (GB21301, Servicebio, China) were incubated at 37 °C for 30 min. Finally, after incubation of DAPI (G1012, Servicebio, China) at room temperature for 10 min, the anti-fluorescence attenuation tablets were sealed. Fluorescence microscope (OlyVIA, OLYMPUS, Japan) was used for observation and photography.

### Real-time quantitative polymerase chain reaction (RT-qPCR)

Mouse brain tissues were collected and total RNA was extracted using Molpure Cell/Tissue total RNA kit (19221ES50, YEASEN, China), and total RNA was reversed into cDNA using PrimeScript RT reagent kit (RR047A, Takara, China). TB Green Premix Ex Taq Ⅱ (Tli RNaseH Plus) (RR820A, Takara, China) was used to perform a PCR reaction with primers of each gene. The specific primer sequences were shown in Table 1. The relative mRNA expression levels of each gene were calculated by 2^−△△Ct^ with β-actin as the internal reference.

**Table 1:** The primer sequences used in the study

	Forward primer	Reverse primer
β-actin	CTACCTCATGAAGATCCTGACC	CACAGCTTCTCTTTGATGTCAC
BDNF	TGACGACGACATCACTGGCTGACACT	TGCCGCTGTGACCCACTCGCTAAT
COX2	AAGTTGATAACCGAGTCGTTCT	GGATGGCATCAGTTTTAAGTCC
CX3CL1	CAGAGCATTGGAAGTTTTGAGG	AGGAGCTAGATCCAGATTGGTA
CX3CR1	TCTGTTGGTGGTCCTCGCTCTC	AGGTAGTGAGTCCAGAAGGGCAAG
DAP12	CTGACTGTGGGAGGATTAAGTC	AGTCTCAGCAATGTGTTGTTTC
FXR1	GACGAAGTTGATGCTTATGTCC	TTCCGGTGTCTTCATCTAACTC
FXR2	GCTTGAGTATCACCTCTCCTAC	GGAGCTTTCGTCTGTGGTATAG
GluA2	AACAAATGGTGGTACGACAAAG	TGTAGAATACTCCAGCAACGTT
GluN2B	AAGAAGAATCGGAACAAACTGC	CAGCTGGCATCTCAAACATATG
IL-1β	CACTACAGGCTCCGAGATGAACAAC	TGTCGTTGCTTGGTTCTCCTTGTAC
iNOS	ATCTTGGAGCGAGTTGTGGATTGTC	TAGGTGAGGGCTTGGCTGAGTG
NGF	TCAGACACTCTGGATCTAGACT	CTGTTGTTAATGTTCACCTCGG
PSD-95	ATGTGCTTCATGTAATTGACGC	TTTAACCTTGACCACTCTCGTC
TDP2	GTGGTTTACCTGACAACGTTTT	GGATCCTGAGGTTGTTATTTGC

### Western blot analysis (WB)

WB was used to detect the expression of the short-chain fatty acid (SCFA) receptor Olfr78 in the hippocampus. The protein lysate was obtained from the hippocampus by RIPA (P0013, Beyotime, China), quantitated and denatured, and then separated by SDS-PAGE and transferred to the PVDF membrane. After sealing with 5% skim milk, the primary antibody Olfr78 (PA5-75357, Invitrogen, USA) and β-actin (AC026, abclonal, China) were incubated at 4℃ overnight, and the secondary antibody (S0001, Affinity biosciences, China) was incubated at room temperature for 2 h. After the reaction, the color reaction was carried out by ECL (17046, zen-bio, China) and Tanon fluorescence image analysis system (5200 Multi, Tanon, China), and the relative protein expression of Olfr78 was calculated.

### 16 s rRNA V3 + V4 high throughput sequencing

Colonic contents were collected aseptically, total DNA was extracted, quantified and measured by Nanodrop and 1.2% agar-gel electrophoresis. The target sequences that can reflect the composition and diversity of bacterial flora, such as microbial ribosomal RNA or specific gene fragments, were taken as targets, corresponding primers were designed according to the conserved regions in the sequences, and specific Barcode sequences were added to the samples, and then PCR amplification was performed on the variable regions of rRNA genes or specific gene fragments. The amplified products were purified by magnetic beads, and fluorescence quantification was performed using a quant-iT picoGreen dsDNA assay kit (P7589, Invitrogen, USA) and a microplate reader (FLx800, BioTek, USA). According to the fluorescence quantitative results, each sample was mixed according to the corresponding proportion according to the sequencing quantity requirement of each sample. TruSeq Nano DNA LT Library Prep Kit (Illumina, USA) was used to prepare sequencing libraries and perform high-throughput sequencing. The original data were collected and the results were analyzed.

### Determination of SCFA content

Acetic acid, propionic acid, butyric acid, isobutyric acid, valeric acid, isovaleric acid, and caproic acid were used to prepare the standard stock solutions. In addition, blood samples of mice in each group were collected and centrifuged at 3000 rpm for 15 min. Appropriate serum was collected and added with 50 µL 15% phosphoric acid, 100 µL 125 µg/mL internal standard solution (isocaproic acid), and 400 µL ether, mixed for 1 min, centrifuged at 12,000 rpm at 4 ℃ for 10 min, and supernatant was obtained for detection. The Gas chromatography analysis was performed under the trace 1300 gas chromatograph (Thermo Fisher Scientific, USA) with Agilent HP-INNOWAX capillary column (30 m × 0.25 mm ID × 0.25 μm) and helium was used as the carrier gas at 1 mL/min. The sample size was 1 µL and the shunt ratio was 10:1. The injector temperature is 250℃, the ion source temperature is 300℃, and the transmission Line temperature is 250℃. The column temperature was programmed to increase from an initial temperature of 90 ℃, followed by an increase to 120 ℃ at 10 ℃/min, and to 150 ℃ at 5 ℃/min, and finally to 250 ℃ at 25 ℃/min which was Maintained for 2 min. Mass spectrometry was performed by ISQ 7000 mass spectrometer (Thermo Fisher Scientific, USA) with electron impact ionization mode. Single ion monitoring (SIM) mode was used with an electron energy of 70 eV.

### Statistical analysis

All experimental data were analyzed using SPSS software, and finally expressed as mean ± standard deviation. All data were normally distributed. Independent sample t-test was used for differential expression between the two groups, and one-way analysis of variance was used for differential expression between the multiple groups. *P* < 0.05 was considered to have a significant difference in statistical results.

## Results

### Pg regulated blood glucose, lipids, and cognitive impairment in T2DM mice

**Fig. 2:**
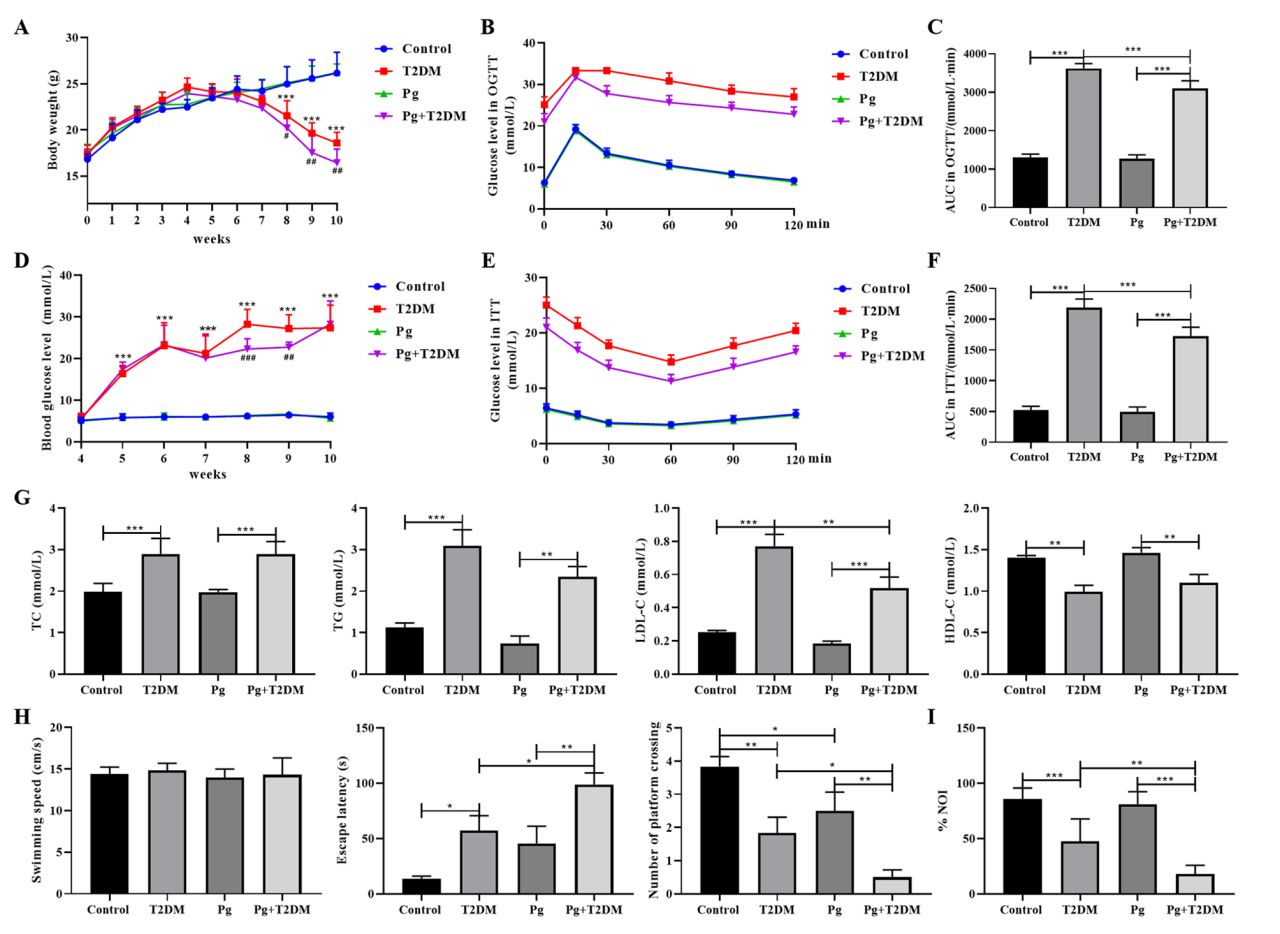
Effects of Pg on blood glucose, lipids and cognitive impairment in T2DM mice. **A**: Weight changes in mice during the experiment. **B**: Oral glucose tolerance test (OGTT) in mice. **C**: Area under curve (AUC) in OGTT. **D**: Changes in random blood glucose levels of mice during the experiment. **E**: Mouse insulin tolerance test (ITT). **F**: The AUC in ITT. **G**: TC, TG, LDL-C, and HDL-C levels of mice at the end of the experiment. **H**: The average swimming speed, escape latency of mice, and the number of times the mice crossed the platform in the Morris water maze experiment. **I**: The novel object recognition index (NOI) of mice in new object recognition experiment. *n* = 6, *P < 0.05, **P < 0.01, ***P < 0.001

During the experiment, the weight of the mice was monitored every week. As shown in Fig. 2A, the weight of mice in the T2DM/Pg + T2DM group decreased significantly from the 8th week (*P* < 0.001). There was no difference in body weight between Pg and control group (*P* > 0.05), but the body weight of mice in the Pg + T2DM group also decreased significantly from the 8th week compared with the T2DM group (*P* < 0.05), indicating that Pg only affected body weight of T2DM mice. In addition, blood glucose monitoring of mice showed that the blood glucose of mice in the T2DM/Pg + T2DM group was significantly higher than that in the control/Pg group (*P* < 0.001, Fig. 2D). Pg did not affect the random blood glucose level of control mice (*P* > 0.05), but significantly reduced the random blood glucose level of mice in the T2DM group from the 8th week (*P* < 0.01, Fig. 2D). Similarly, the OGTT results showed that the blood glucose levels of the model mice significantly increased after glucose intake, and the decline rate was slow (Fig. 2B). Even after 120 min, the levels were still higher than the normal level, with a significant increase in AUC (*P* < 0.001, Fig. 2C), indicating impaired glucose tolerance. Pg significantly reduced the AUC of the model mice, and the glucose tolerance of the model mice improved (*P* < 0.001, Fig. 2B and C). The ITT results also showed that the blood glucose levels of the model mice decreased slowly after insulin stimulation, with a significant increase in AUC, and insulin sensitivity decreased (*P* < 0.001, Fig. 2E and F). Pg significantly reduced the AUC of the model mice and enhanced their insulin sensitivity (*P* < 0.001, Fig. 2E and F). Compared with the control/Pg group, the levels of TC, TG, and LDL-C in the T2DM/Pg + T2DM group were significantly increased, and the levels of HDL-C were significantly decreased (*P* < 0.001, Fig. 2G). Pg did not affect lipid levels in control mice (*P* > 0.05, Fig. 2G), but reduced LDL-C levels in the T2DM mice (*P* < 0.01, Fig. 2G). Overall, Pg did not affect normal mice and only had a partial effect on glycolipid metabolism in T2DM mice.

Further behavioral tests of the mice showed no difference in average swimming speed between the groups during the water maze experiment (*P* > 0.05, Fig. 2H). In the test stage, the escape latency of mice in the T2DM/Pg + T2DM group was significantly increased compared with the control/Pg group (*P* < 0.05, Fig. 2H), and the number of times crossing the platform was also significantly reduced (*P* < 0.01, Fig. 2H), indicating that the spatial learning ability of mice was impaired by T2DM modeling. Pg treatment further increased the escape latency of mice in the T2DM group and reduced the number of mice passing through the platform (*P* < 0.05, Fig. 2H), indicating that Pg was involved in the spatial learning impairment caused by T2DM. In addition, the results of the new object recognition experiment showed that the NOI of mice in the T2DM/Pg + T2DM group was significantly lower than that in the control/Pg group (*P* < 0.001, Fig. 2I). Pg had no effect on the NOI of normal mice (*P* > 0.05), but it would further reduce the NOI of T2DM mice (*P* < 0.01, Fig. 2I). Water maze and new object recognition experiments confirmed the cognitive impairment in T2DM mice, and Pg could further aggravate the cognitive impairment caused by T2DM.

### Pg promoted hippocampal neuron injury in T2DM mice

**Fig. 3:**
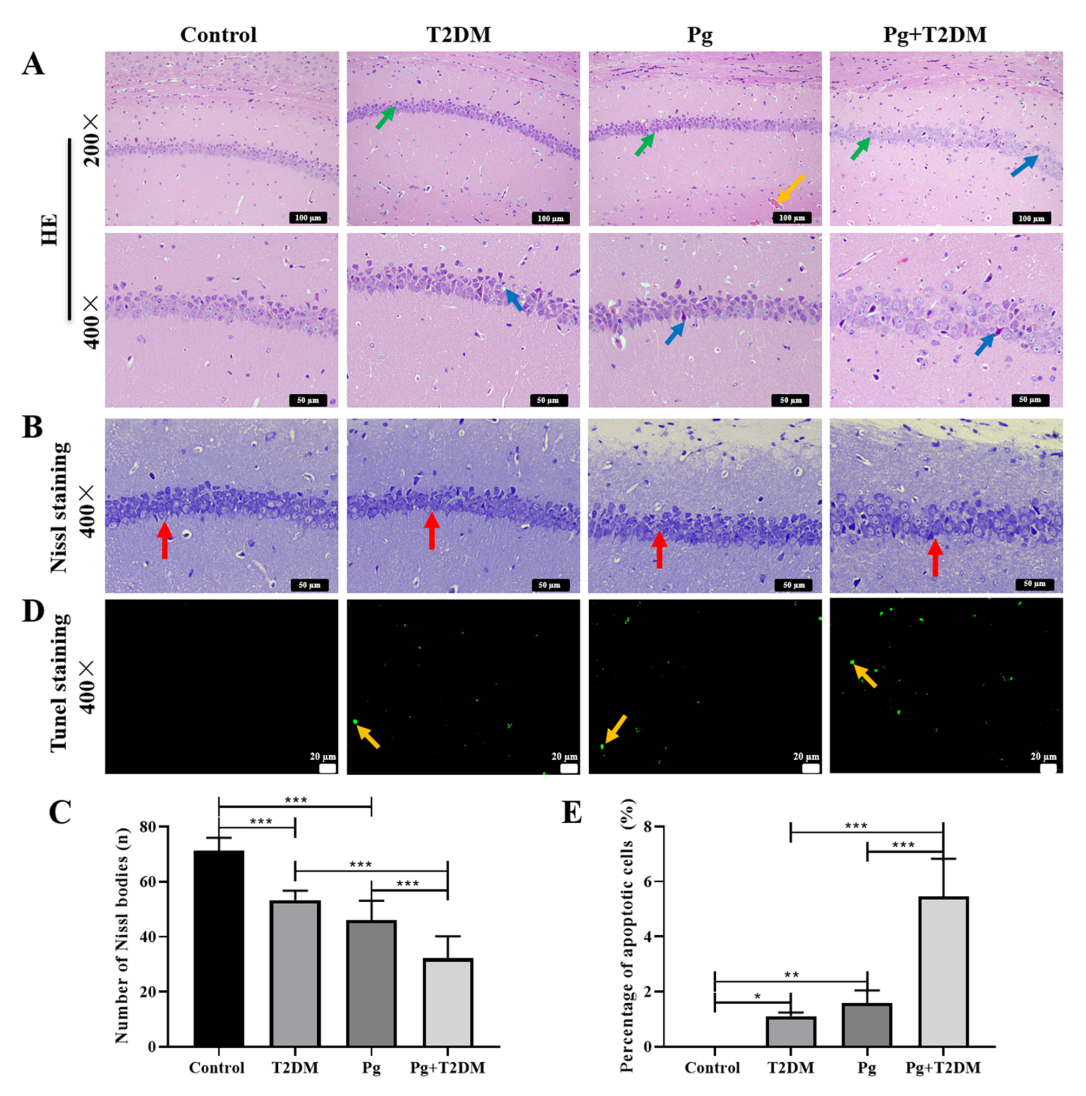
Effects of Pg on hippocampal neuron injury in T2DM mice. **A**: HE staining was used to observe the morphological and organizational changes of neurons in the CA1 region of the hippocampus. Magnification: 200× and 400×. The green arrows point to the dark-colored neurons, the yellow arrows point to the capillaries, and the blue arrows point to the vertebral cells. **B**, **C**: Nissl staining was used to calculate the number of Nissl bodies in the CA1 region of the hippocampus. Magnification: 400×. The red arrows point to the Nissl bodies. **D**, **E**: TUNEL staining was used to observe neuronal apoptosis in the CA1 region of the hippocampus. Magnification: 400×. The yellow arrows point to apoptotic cells. *n* = 6, ^*^ *P* < 0.05, ^**^ *P* < 0.01, ^***^ *P* < 0.001

Further analysis of the damage of mice hippocampal neurons showed that dark-colored neurons were observed in the hippocampal region of brain tissue of T2DM mice, and pathological changes such as vacuolar degeneration, necrosis, and loose arrangement of vertebral cells were observed (Fig. 3A). Compared with T2DM mice, the lesions of the Pg + T2DM group were more severe (Fig. 3A). Toluidine blue staining results showed that compared with the control group, the number of Nissl bodies in the hippocampus of mice in T2DM and Pg groups was significantly decreased (*P* < 0.001), and the number of Nissl bodies in the Pg + T2DM group was further reduced compared with the T2DM group (*P* < 0.001, Fig. 3B and C). In addition, TUNEL staining showed that the percentage of apoptotic cells in the hippocampal tissue of mice in both T2DM and Pg groups was significantly increased (*P* < 0.05), and the percentage of apoptotic cells in the Pg + T2DM group was further increased than that in the T2DM group (*P* < 0.001, Fig. 3D and E). These results suggested that the hippocampal neurons of T2DM mice have obvious damage, and Pg aggravates the damage caused by T2DM.

### Pg reduced hippocampal synaptic plasticity in T2DM mice

**Fig. 4:**
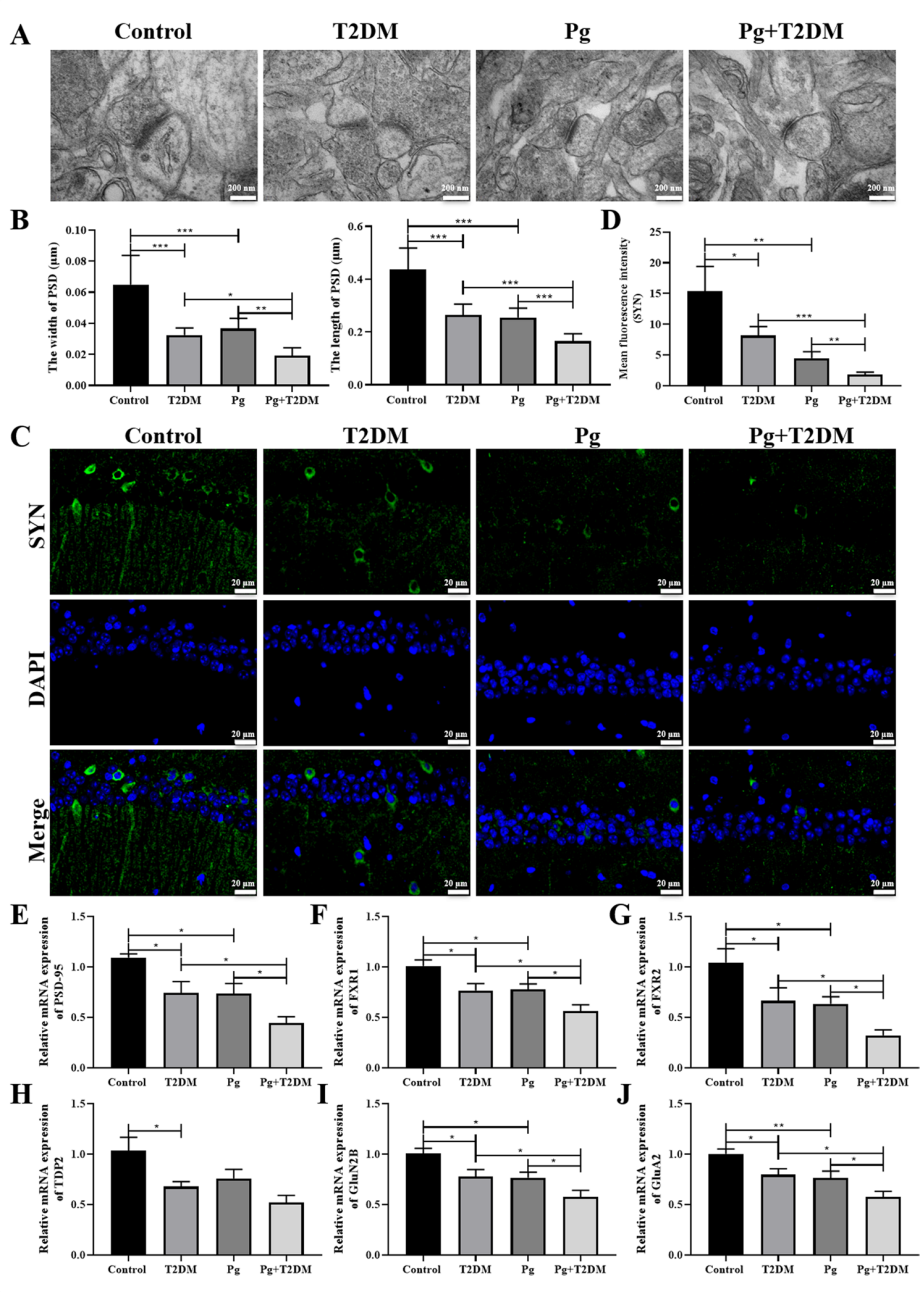
Effects of Pg on hippocampal synaptic plasticity in T2DM mice. **A**: The ultrastructure of hippocampal neurons and synapses was observed by transmission electron microscopy. Magnification: 100,000×. **B**: Width and length of postsynaptic density (PSD). **C**, **D**: The expression of SYN in the CA1 region of the hippocampus was observed by immunofluorescence staining. Magnification: 400×. The expressions of PSD-95 (**E**), FXR1 (**F**), FXR2 (**G**), TDP2 (**H**), GluN2B (**I**), GluA2 (**J**) were detected by RT-qPCR. *n* = 6, ^*^ *P* < 0.05, ^**^ *P* < 0.01, ^***^ *P* < 0.001

The ultrastructure of hippocampal neurons and synapses was observed by transmission electron microscopy (Fig. 4A), and the width and length of PSD were measured (Fig. 4B). Results showed that compared with the control group, the width and length of hippocampal PSD in the T2DM and Pg groups were significantly reduced (*P* < 0.001), and the PSD width and length in the T2DM/Pg group were further reduced in the Pg + T2DM group (*P* < 0.001, Fig. 4B). Immunofluorescence analysis of SYN expression also showed that SYN expression was significantly decreased in the T2DM and Pg groups, and Pg + T2DM further decreased SYN expression in the T2DM/Pg groups (*P* < 0.05, Fig. 4C and D). Further detection of related genes by RT-PCR showed that the expressions of PSD-95 (Fig. 4E), fragile X-related 1 (FXR1, Fig. 4F), FXR2 (Fig. 4G), GluN2B (Fig. 4I) and GluA2 (Fig. 4J) in the brain tissue of mice in T2DM and Pg groups were significantly decreased (*P* < 0.05), and Pg + T2DM further decreased the expression of these genes in the brain tissue of mice in the T2DM/Pg group (*P* < 0.05). These results suggested that hippocampal synaptic plasticity was significantly decreased in both T2DM and Pg groups, and Pg + T2DM could further reduce synaptic plasticity in T2DM mice.

### Pg aggravated hippocampal microglial inflammation in T2DM mice

**Fig. 5:**
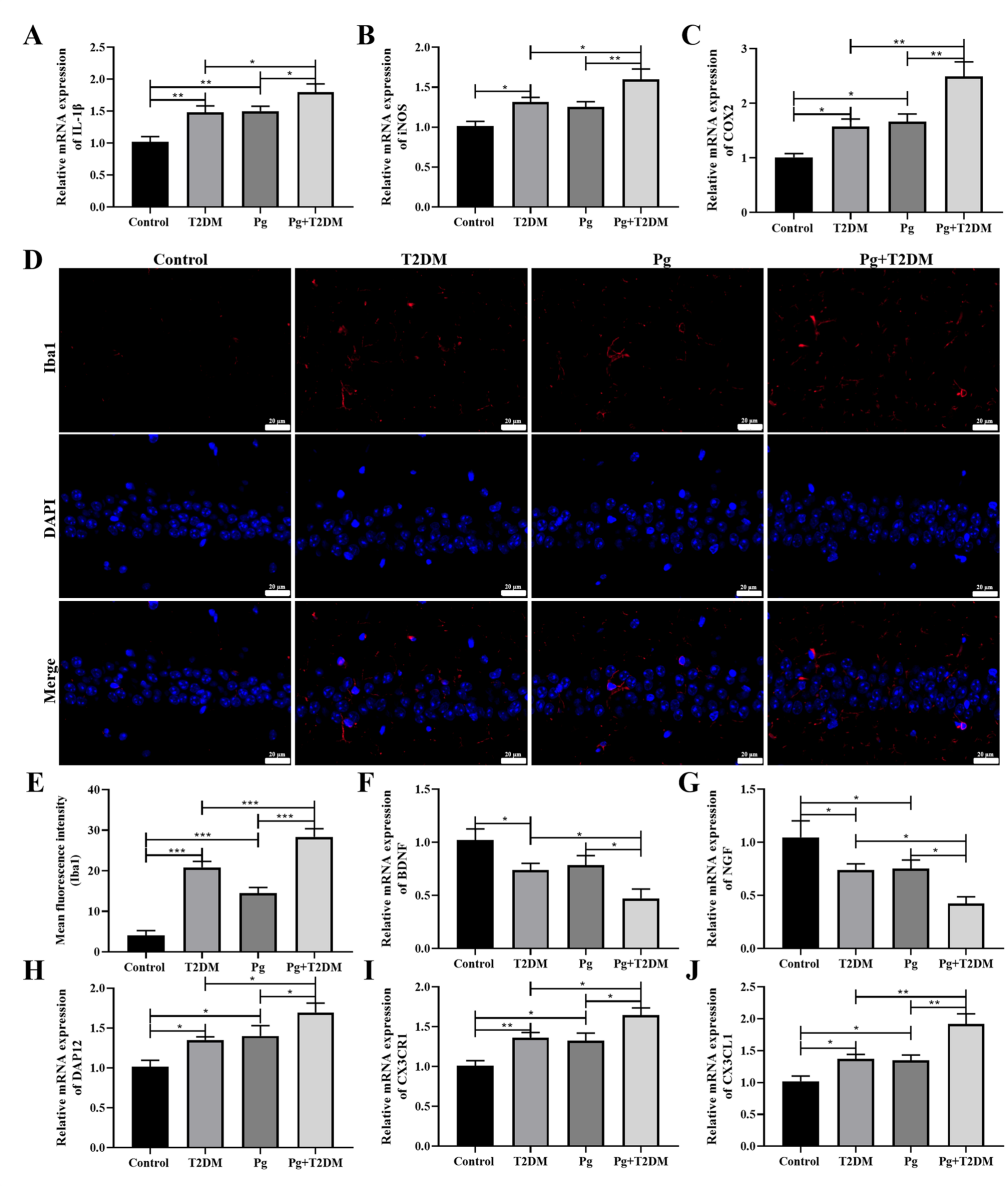
Effects of Pg on hippocampal microglia inflammation in T2DM mice. The expression of neuroinflammatory cytokines IL-1β (**A**), iNOS (**B**), and COX2 (**C**) were detected by RT-qPCR. **D**, **E**: Immunofluorescence staining with Iba1 was used to observe the activation of microglia in the CA1 region of the hippocampus. Magnification: 400×. Microglia-neuron interaction genes BDNF (**F**), NGF (**G**), DAP12 (**H**), CX3CR1(**I**), and CX3CL1(**J**) were detected by RT-qPCR. *n* = 6, ^*^ *P* < 0.05, ^**^ *P* < 0.01, ^***^ *P* < 0.001

The effects of Pg on the inflammatory response of microglia in T2DM mice were further analyzed. The expression of neuroinflammatory factors in brain tissue was examined by RT-PCR, and it was found that the expressions of IL-1β, iNOS, and COX2 in brain tissue of T2DM mice were significantly increased (*P* < 0.05), and the expression of inflammatory factors in brain tissue of Pg + T2DM group was further increased compared with that of T2DM/Pg group (*P* < 0.05, Fig. 5A-C). Immunofluorescence detection of Iba1 was performed to analyze the activation of microglia. It was found that the expression of Iba1 in the hippocampus of mice in both T2DM and Pg groups was significantly increased, and the expression of Iba1 was further increased in the Pg + T2DM group (*P* < 0.001, Fig. 5D and E). The expressions of microglia-neuron interaction genes BDNF (Fig. 5F), NGF (Fig. 5G), DAP12 (Fig. 5H), CX3CR1 (Fig. 5I) and CX3CL1 (Fig. 5J) showed that the expression of BDNF in the T2DM group was significantly decreased (*P* < 0.05), and Pg had no significant regulatory effect on BDNF in the control group (*P* > 0.05, Fig. 5F). And the expression of NGF was significantly decreased in both T2DM and PG groups, while the expressions of DAP12, CX3CR1, and CX3CL1 were significantly increased (*P* < 0.05, Fig. 5F-J). The Pg + T2DM group further regulates the expression of these genes in the T2DM and Pg groups (*P* < 0.05, Fig. 5F-J). These results indicated that Pg significantly promoted the activation of hippocampal microglial inflammatory response induced by T2DM.

### Pg regulated gut microbiota and metabolites in T2DM mice

**Fig. 6:**
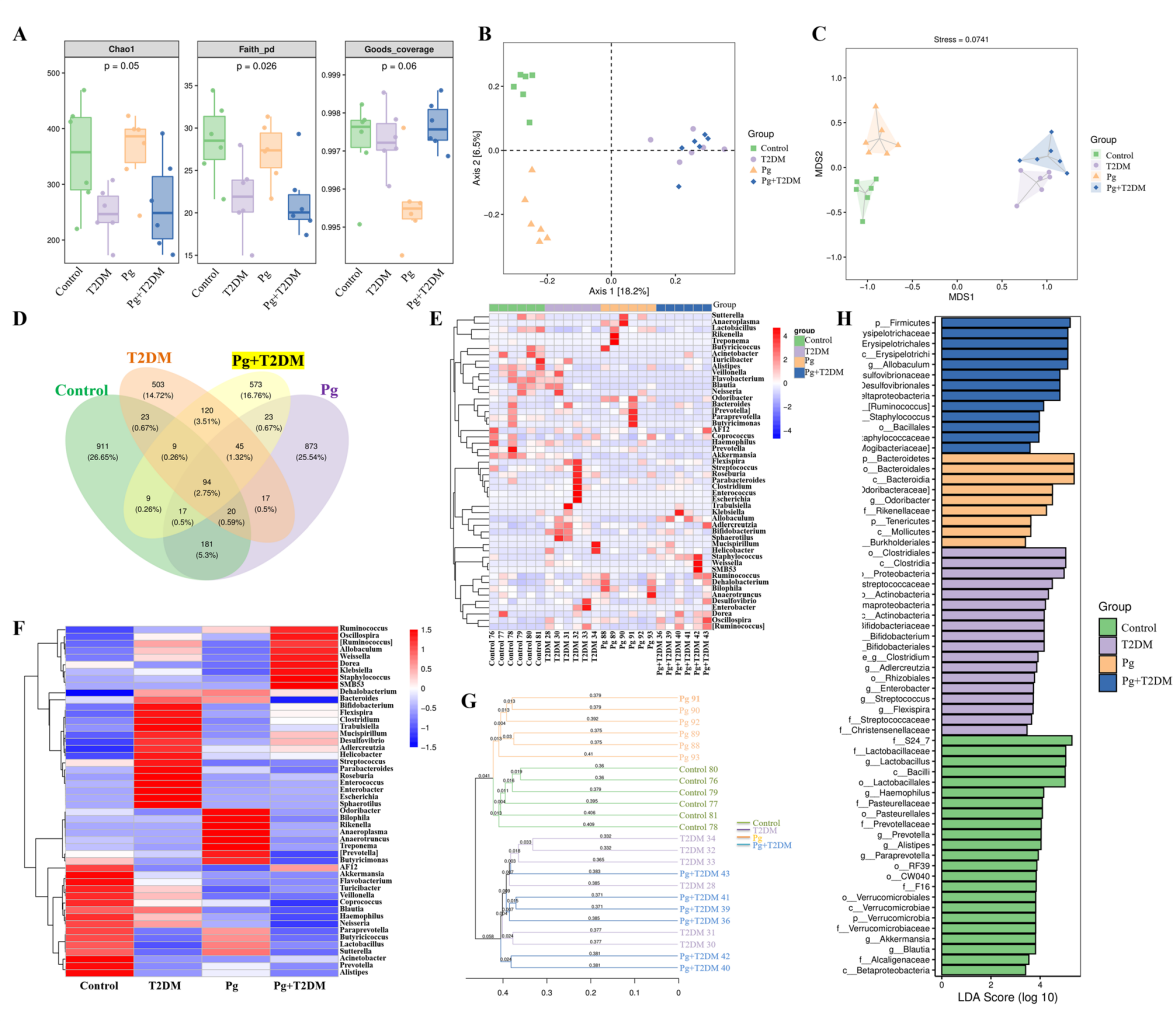
Effects of Pg on gut microbiota of T2DM mice. **A**: Grouping box plot of α diversity index. **B**: Two-dimensional ordering diagram of samples analyzed by PCoA. **C**: NMDS 2D sorting diagram. **D**: Venn diagram of groups. **E**: Generic level composition heat map of species clustering. **F**: Heat map of each group. **G**: UPGMA clustering tree based on sample distance matrix. **H**: Histogram of LDA effect size for species. *n* = 6

Studies have shown that the gut microbiota of T2DM patients is disordered, and the disordered gut microbiota can affect cognitive levels through the gut-brain axis. Pg may also further cause gut microbiota disturbance in T2DM, thereby mediating the gut-brain axis effect on cognition. We analyzed the gut microbiota of mice in each group through 16 s rRNA sequencing, as shown in Fig. 6. The analysis results of α diversity were shown in Fig. 6A, richness was represented by the Chao1 index, evolution-based diversity was represented by Faith’s PD index, and coverage was represented by Good’s coverage index. And Jaccard-based PcoA was used to demonstrate beta diversity (Fig. 6B). Non-dimensional multidimensional scaling analysis (NMDS) also simplifies the data structure through dimensionality reduction decomposition of the sample distance matrix, so as to describe the distribution characteristics of samples at a specific distance scale. Jaccard-based NMDS was shown in Fig. 6C. Both α and β diversity indicated that there were some differences in microbial communities among samples of each group. To investigate which species are common and which are unique among different groups, community analysis was performed using the Venn diagram, as shown in Fig. 6D. The species composition differences among samples were further compared, and the clustering tree ranking was drawn according to the correlation between samples, that is, the clustering heat map was drawn, as shown in Fig. 6E and F. In addition, the similarity between samples is displayed in the form of hierarchical tree, and the clustering effect is measured by the branch length of the clustering tree, the Jaccard-based clustering tree as shown in Fig. 6G. LEfSe analysis is an analytical method that combines non-parametric Kruskal-Wallis and Wilcoxon rank sum tests with linear discriminant analysis effect size, which can directly analyze the difference of all classification levels at the same time. The histogram of the LDA value distribution of different species in this study was shown in Fig. 6H.

**Fig. 7:**
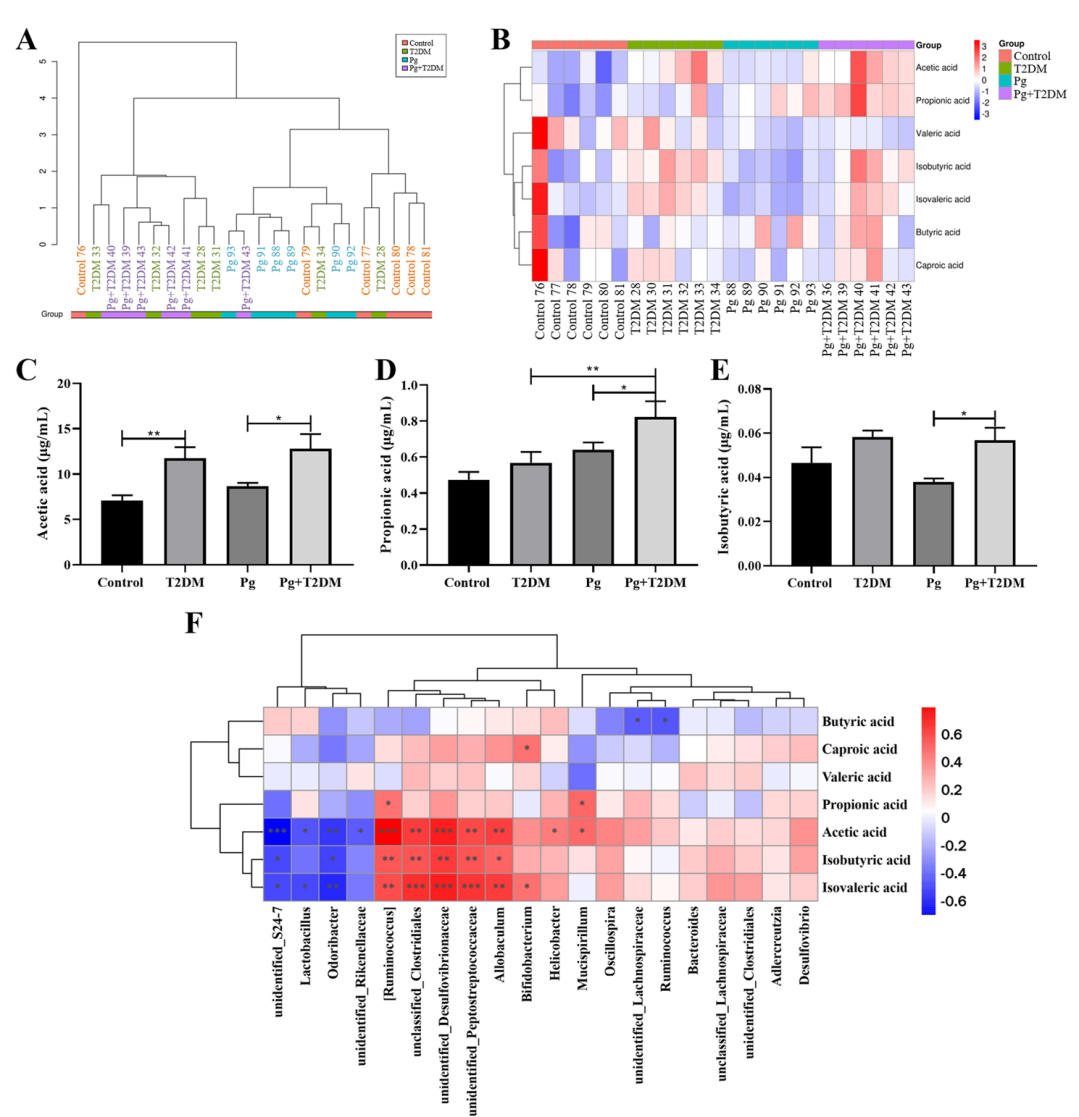
Effects of Pg on SCFAs in T2DM mice. The content of SCFAs in the serum of mice was detected by high-performance liquid chromatography. **A**: Sample level clustering tree. **B**: Aggregate metabolite clustering heat map. **C**: Quantitative results of acetic acid. **D**: Propionic acid content. **E**: Isobutyric acid content. **F**: The correlation between the intestinal microbiota at the genus level and SCFAs. *n* = 6, ^*^ *P* < 0.05, ^**^ *P* < 0.01, ^***^ *P* < 0.001

**Fig. 8:**
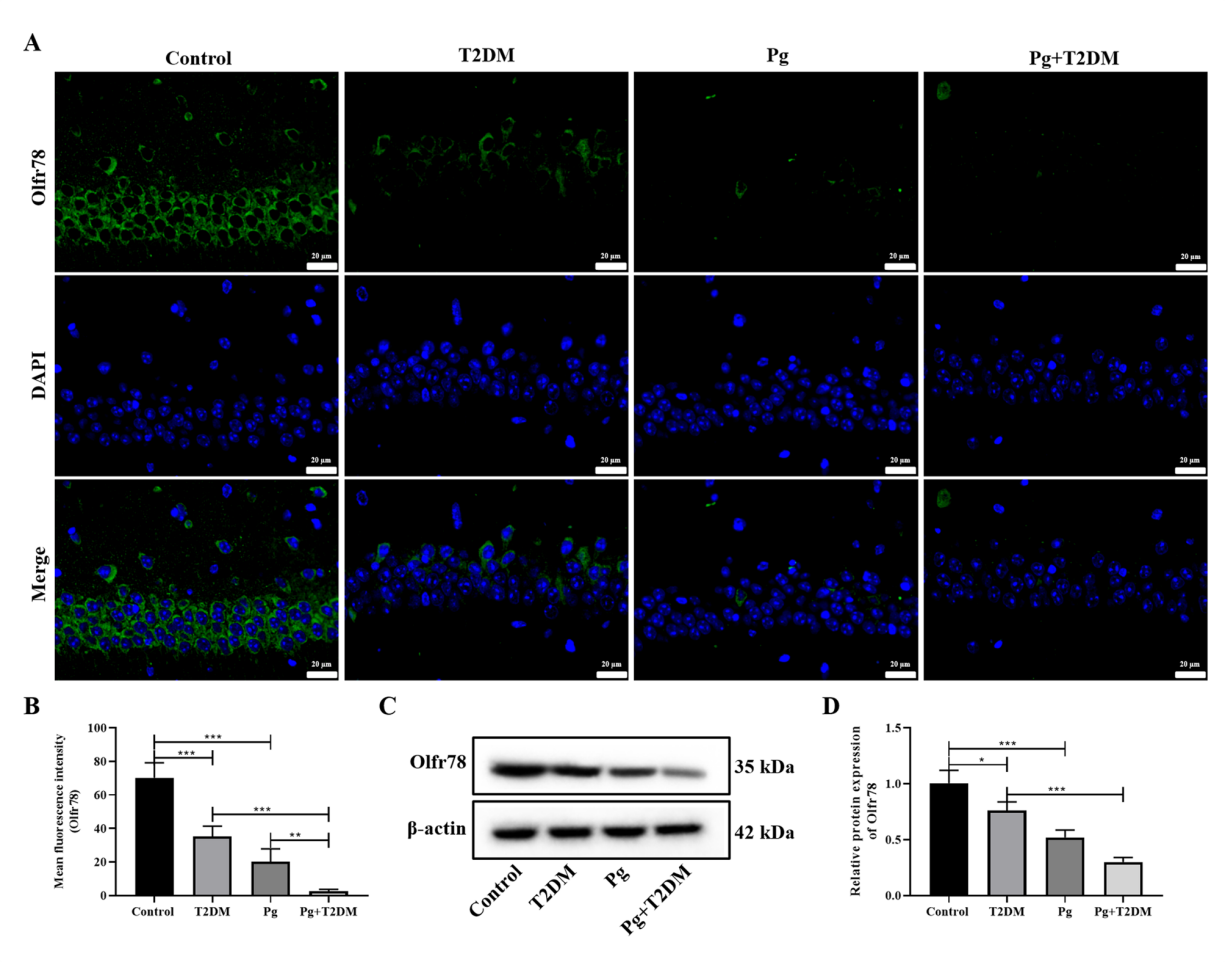
Effects of Pg on SCFA receptor in T2DM mice. **A**: The expression of SCFA receptor Olfr78 in the hippocampus was detected by immunofluorescence. Magnification: 400×. **B**: The mean fluorescence intensity of Olfr78. **C**: WB was used to detect the expression of Olfr78 in the hippocampus. **D**: The relative protein expression level of Olfr78. *n* = 6, ^*^ *P* < 0.05, ^**^ *P* < 0.01, ^***^ *P* < 0.001

In addition, the serum metabolomics was further analyzed by targeting SCFAs. The sample level clustering tree was shown in Fig. 7A, and the overall metabolite clustering heat map was shown in Fig. 7B. Analysis results of specific differential metabolites in serum metabolomics were shown in Fig. 7C-E, mainly involving acetic acid, propionic acid, and isobutyric acid. A further analysis of the correlation between SCFAs and the intestinal microbiota was conducted, and the results are shown in Fig. 7F. Acetic acid, isobutyric acid, and isovaleric acid have significant correlations with various gut microbiota, including unidentified_S24-7, Lactobacillus, Odoribacter, [Ruminococcus], unclassified_Clostridiales, unidentified_Desulfovibrionaceae, unidentified_Peptostreptococcaceae, Allobaculum, and Bifidobacterium, etc. In addition, the expression of the SCFA receptor Olfr78 in the hippocampus of mice was further analyzed by immunofluorescence. The results showed that both T2DM and Pg significantly reduced the expression of Olfr78 in the hippocampus of control mice, and Pg could further reduce the expression of Olfr78 in the hippocampus of T2DM mice (*P* < 0.01, Fig. 8A and B). The results of WB were similar to those of immunofluorescence (Fig. 8C and D), suggesting that Pg plays a role in the regulation of the SCFA metabolism pathway in T2DM.

## Discussion

Diabetes is the most common serious metabolic disease in humans and is characterized by abnormal blood glucose due to defective insulin secretion or insulin resistance(Refardt et al. 2020). T2DM is strongly associated with cognitive impairment and has a higher risk of developing cognitive impairment compared to the general population(You et al. 2021). As a complication of T2DM, diabetic cognitive impairment is easily transformed into AD with the development of the disease course(Michailidis et al. 2022). Meanwhile, hyperglycemia is also considered to be one of the important pathogenesis factors of AD(Chakrabarty et al. 2022). Clinical and epidemiological studies have shown that T2DM patients are at twice the risk of developing AD than non-T2DM patients(Rojas et al. 2021). T2DM-related cognitive dysfunction is mainly characterized by the decline of memory, learning ability, and psychomotor efficiency(Ehtewish et al. 2022). This study found that T2DM model mice had abnormal blood glucose and lipids, and showed impaired memory function, reduced exploration ability, and obvious cognitive impairment. In addition, Pg could further promote the dysglycemia of T2DM mice and further impair the cognitive ability of T2DM mice.

Pg has been recognized as a major pathogen of oral disease periodontitis with a variety of pathogenic factors, including its structural components lipopolysaccharide, pili, heat shock protein, and secretory components(Mysak et al. 2014). These factors stimulate adjacent epithelial cells to produce various cytokines, leading to local tissue destruction, disrupting the host antimicrobial defenses, and leading to overgrowth and spread of symbiotic bacteria in the mouth and intestines(Gasmi Benahmed et al. 2022). Pg is also a facultative intracellular bacterium that can invade a variety of host cells and participate in the formation and development of a variety of systemic diseases(Li et al. 2022). Previous studies have confirmed that mice given Pg have abnormal glucose tolerance, insulin resistance, and higher intramuscular adipose tissue content damage, which is related to systemic endocrine status(Watanabe et al. 2021). In addition, Pg can penetrate the blood-brain barrier or nerve diffusion into and remain in the brain, and is associated with causing the corresponding damage of the nervous system(Yu et al. 2021), which is considered to be a high-risk pathogenic factor for AD(Kanagasingam et al. 2020). This study found that the spatial learning and memory ability of T2DM mice was further impaired after Pg treatment, mainly manifested as the escape latency increased and the number of platform crossing decreased. The NOI was significantly reduced in T2DM mice treated with Pg, suggesting that Pg further impaired the short-term memory ability of T2DM mice. Overall, Pg further aggravated cognitive dysfunction in T2DM mice.

T2DM and AD have similar pathological changes, and the hippocampus is one of the main areas of brain stem structural changes in the early stage of T2DM(Lee et al. 2021). As an important factor in cognitive performance, hippocampal neurons and synaptic plasticity play an important role in reflecting learning and memory ability(Ambrogioni and Ólafsdóttir 2023). Memory deficits and autonomic behavior abnormalities are considered to be two important manifestations of hippocampal damage caused by T2DM(Li et al. 2020; Zhang et al. 2021). This study showed that compared with the control group, neurons in the hippocampal CA1 region of mice in the T2DM, Pg and Pg + T2DM groups were all damaged, with obvious loss of neurons and significantly increased proportion of neuron apoptosis, among which the hippocampal neuron damage in the Pg + T2DM group was relatively heaviest. These results confirmed the sensitivity and vulnerability of the hippocampus to blood glucose disorders, and also confirmed that cognitive dysfunction is closely related to changes in the structure and number of neurons(Henn et al. 2022), and Pg can aggravate the cognitive dysfunction associated with T2DM by promoting the damage of hippocampal neurons in T2DM mice. In addition, further experiments confirmed that there was obvious synaptic damage in the hippocampus of T2DM mice, and Pg further reduced the length and width of PSD, the expression of SYN protein, and the mRNA expressions of PSD-95, FXR1, FXR2, GluN2B, and GluA2. SYN is an important synaptic vesicle protein in neurons and is an important substance involved in cell membrane structure and other components(Burré et al. 2018). PSD-95 is an enriched protein in the excitatory postsynaptic dense region, which is involved in the regulation of glutamatergic synaptic plasticity through interaction with NMDA receptors and AMPA receptors(Levy et al. 2022). GluN2B, an NMDA receptor, and GluA2, an AMPA receptor, mediate rapid excitatory synaptic transmission in the CNS and are involved in the regulation of learning and memory activities(Fukata et al. 2021). The FXR family, including FXR1/FXR2, also plays a key role in neuronal plasticity(Majumder et al. 2020). Our results confirmed that synaptic plasticity was weakened in T2DM mice, and Pg further promoted the effects of T2DM on synaptic plasticity in mice. In addition, the expressions of neuroinflammatory factors in brain tissue of T2DM mice were significantly increased, hippocampal microglia were activated, the expressions of BDNF and NGF were decreased, the expressions of DAP12, CX3CR1, and CX3CL1 were increased, and Pg further promoted the inflammatory response of hippocampal microglia in T2DM mice. In physiological or pathological states, the regulation of neurons by microglia is crucial for maintaining brain homeostasis(Marinelli et al. 2019). In addition to the surface receptors of microglia, microglia can regulate neurons through a series of molecules, such as neurotransmitters, purine and adenosine signaling molecules, cytokines (such as neurotrophic factors NGF, BDNF, and CX3CL1/CX3CR1)(Umpierre and Wu 2021). The cytokines released by microglia participate in the regulation of neuronal function and play neuroprotective or harmful roles(Cserép et al. 2021). Our results suggested that hippocampal neuron injury, synaptic plasticity changes, and microglial inflammation in T2DM may be the causes of cognitive impairment, and Pg further promoted cognitive impairment in T2DM mice by aggravating these processes.

Gut microbes play an important role in immune, metabolic, and nervous system development and function(Liu et al. 2023). Studies have shown that gut microbiota is closely related to T2DM, and may be a new target for the treatment of T2DM and related diseases(Liu et al. 2022). Pg, as the main periodontal pathogenic bacteria, has strong acid resistance and can resist various factors to survive in the intestine(Chen et al. 2022). The anaerobic and high pH environment of the colon mucosa is more conducive to the adhesion of Pg(Simas et al. 2021). Once the flora is disturbed, harmful bacteria occupy a dominant position, which will cause the intestinal immune state and intestinal barrier function of the host, and toxic substances such as bacterial toxins and metabolites may enter the systemic circulation through the intestine, resulting in damage to distant tissues and organs of the body(Kim and Jazwinski 2018). This study also showed that T2DM mice had obvious gut microbiota disturbance, and Pg further caused gut microbiota disturbance in T2DM mice. In addition, serum metabolomics results showed that the content of SCFAs in T2DM mice was changed, and the content of SCFAs was significantly correlated with the changes in various gut microbiota. At the same time, the expression of Olfr78 in the hippocampus of T2DM mice was significantly decreased, and Pg also decreased the expression of Olfr78 in the hippocampal tissues of both control and T2DM mice. Olfr78, a SCFA receptor, produced from indigestible polysaccharides by gut microbial fermentation, is expressed in intestinal endocrine cells of the colon and plays an important role in the gut microbiota(Nishida et al. 2021). Pg may regulate the expression of Olfr78 through multiple pathways, including the activation of inflammatory signals mediated by virulence factors, the host metabolic reprogramming driven by metabolites, and potential direct receptor interactions. Previous studies have shown that Pg, as an oral pathogenic bacterium, its endotoxin or metabolites may indirectly affect the balance of the intestinal microbiota through the “oral-gut axis“(Lam et al. 2023), and the SCFAs produced by the gut microbiota are the main ligands of Olfr78. Therefore, the metabolites of Pg may directly mimic the structure of SCFAs and bind to Olfr78, or regulate SCFAs to indirectly affect the expression of Olfr78. In addition, Pg infection can activate systemic inflammatory responses(Wang et al. 2024), and the chronic inflammatory environment may inhibit the expression of Olfr78(Kotlo et al. 2020). Overall, we suggested that Pg may affect the cognition function of T2DM mice by mediating the gut-brain axis through the regulation of gut microbiota and its metabolic pathway.

## Conclusion

In conclusion, T2DM mice have significant cognitive impairment, and Pg could aggravate the cognitive impairment of T2DM mice. Further analysis showed that Pg increased neuronal injury, decreased hippocampal synaptic plasticity, and promoted microglial inflammation in T2DM mice. Gut microbiota sequencing and metabolite analysis showed that Pg further cause the disturbance of gut microbiota and its metabolic pathway SCFAs in T2DM mice, thereby mediating the effect of the gut-brain axis on cognition. Pg may be involved in the cognitive impairment of T2DM through gut microbiota and metabolic pathways.

## Data Availability

The datasets used and/or analyzed during the current study are available from the corresponding author on reasonable request.
